# Thermo-Sensitive mPEG-PA-PLL Hydrogel for Drug Release of Calcitonin

**DOI:** 10.3390/gels8050282

**Published:** 2022-05-02

**Authors:** Yu-En Cheng, I-En Wu, Yi-Chen Chen, I-Ming Chu

**Affiliations:** Department of Chemical Engineering, National Tsing Hua University, Hsinchu City 30013, Taiwan; chengyuen@gmail.com (Y.-E.C.); k2258s91@icloud.com (I.-E.W.); yichen961012@gmail.com (Y.-C.C.)

**Keywords:** hydrogel, calcitonin, temperature responses, oral delivery

## Abstract

The oral route is the most popular way of drug administration because of good patient compliance and ease of use. However, the oral delivery of peptides and proteins is difficult, mainly due to poor oral bioavailability. In past decades, researchers have developed several strategies to improve oral bioavailability by avoiding losing activity in the gastrointestinal (GI) tract and enhancing the intestinal permeability of these drugs. Methoxy poly(ethylene glycol)-poly(l-alanine) (mPEG-PA) is a thermo-sensitive hydrogel exhibiting a sol-to-gel phase transition property. This characteristic is appropriate for encapsulating peptide or protein drugs. To enhance the adhesion ability to intestinal mucus, a thermo-sensitive polymer, mPEG-PA, modified with charged amino acid lysine was developed. This positively charged material would help to bind the negatively charged mucin in mucus. The synthesis was conducted by individually synthesizing mPEG-PA and poly(l-lysine) (PLL) of different lengths via ring-opening polymerization. Then, mPEG-PA and PLL were combined using an NHS ester reaction to synthesize the triblock copolymer (mPEG-PA-PLL). Biocompatibility and the release of calcitonin from the synthesized hydrogel particles under different pH were examined. The initial data showed that the newly design material had a promising potential for the oral delivery of peptide drugs.

## 1. Introduction

Oral delivery is the primary drug administration route due to its convenience. However, for macromolecules such as peptides or proteins, oral administration leads to enzymatic degradation and a mucus barrier in the GI tract [1,2,3]. Therefore, protecting peptides and proteins from degradation and increasing their absorption are the vital areas that need to be addressed [4,5]. These kind of efforts can be seen in the development of intestinal patches, intestinal microneedles, particle formulations, permeation enhancers, and ionic liquids [2]. In recent years, some studies have also offered different oral delivery system for peptides and proteins, such as liposome [6], hydrogel [7], lipid carrier [8],and other nanosystems [9].

Hydrogels, with their three-dimensional (3D) network structures, are an excellent candidate for peptides/proteins delivery, where the variation of water absorbency, swelling behavior and stimuli sensitivity can help to control drug release. A lipase enzyme-responsive hydrogel is also a good candidate [10,11]. Hydrogels can confer protection to drugs from enzymatic degradation or low pH in the gastrointestinal (GI) tract [12,13]. Stimuli-sensitive hydrogels can be tailored to meet specific environmental changes and respond by changing their shape or volume [14,15].

Thermo-sensitive hydrogels with a low sol–gel transition property are widely used in drug delivery [16,17], cell scaffolds [18,19,20], and postoperative antiadhesion [21]. In particular, poly(ethylene glycol) (PEG)-polypeptide hydrogels have a good bio-compatibility and provide an in situ gelling ability for easy drug encapsulation [17,22], while poly-l-lysine (PLL) was also found to be useful in drug delivery [23,24]. The sequential nucleophilic substitution reaction has been applied for the preparation of other thermo-sensitive hydrogels [25,26,27].

In this study, a segment of more positively charged PLL is added to the terminal amino acid end of methoxy poly(ethylene glycol)-poly(l-alanine) (mPEG-PA) to obtain a polypeptide triblock copolymer with pH- and temperature-sensitive properties. pH-sensitive hydrogels can exhibit a swelling behavior in response to surrounding external conditions such as ionic strength and pH value [28]. In addition, PLL can help to adhere to GI mucus, a gel-like barrier rich in negative charge on its surface due to its electronic effect [29,30]. Thus, a longer residence time and increased permeability of active compounds may be expected.

Calcitonin produced by parafollicular cells of the thyroid is a 32-amino-acid peptide use to reduce blood calcium (Ca^2+^) [31,32,33]. Synthetic or recombinant calcitonins from different species, such as human, porcine, and salmon, have been used for treatments on hypercalcemia and certain bone problems such as Paget’s disease and postmenopausal osteoporosis. In particular, salmon calcitonin (sCT) is mostly used via injection or nasal spray. Due to the short half-life of sCT in serum, better formulations for prolonged action and bioavailability are needed [34,35].

The synthesis and characterization of a thermo-sensitive triblock copolymer (mPEG-PA-PLL) was undertaken first in this study. mPEG-PA was modified with PLL to increase the electrostatic effect. Then, physicochemical properties of the copolymers in solubilized and hydrogel forms were studied in terms of their assembly and transition in response to temperature or pH change. Peptide release rates and the biocompatibility of the hydrogels were also studied.

## 2. Results and Discussion

### 2.1. The Synthesis and Characterization of mPEG-PA-PLL Triblock Copolymer

**Scheme 1:**
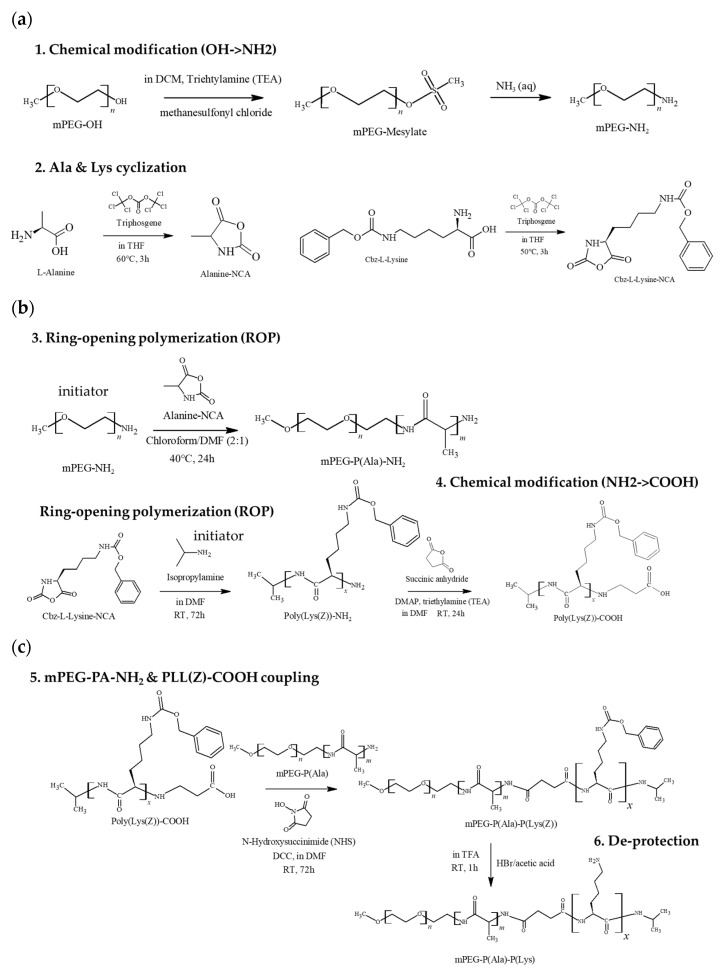
Synthesis scheme of mPEG-PA-PLL. (**a**) Synthesis of mPEG-NH_2_ and the cyclization of Ala and Lys(Z). (**b**) Synthesis of mPEG-PA by ROP and synthesis of PLL(Z)-COOH by ROP and chemical modification. (**c**) Synthesis of mPEG-PA-PLL(Z) by NHS ester coupling reaction and deprotection.

**Figure 1:**
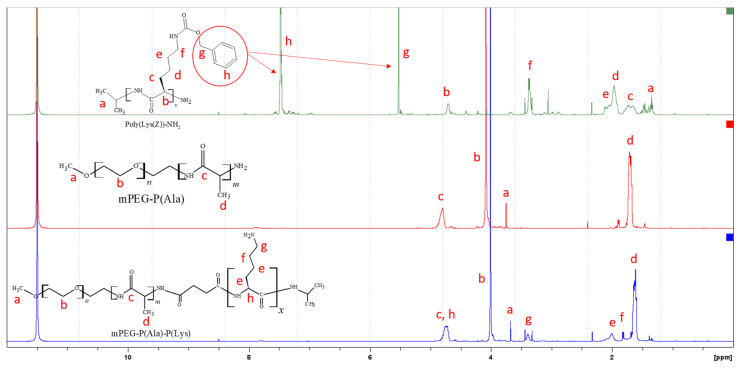
^1^H-NMR spectra of PLL(Z)-NH_2_, mPEG-PA, and mPEG-PA-PLL. a-h were labeled respectively as the chemical structure position corresponding to the peak.

This study focused on mPEG-PA-PLL triblock copolymer for the synthesis and applications. The synthesis route of mPEG-PA-PLL is illustrated in Scheme 1. The mPEG-PA was prepared by ring-opening polymerization (ROP) of l-Alanine-carboxyanhydride (NCA) with mPEG as initiator. The ^1^H NMR spectra of PLL(Z)–NH_2_, mPEG-PA diblock copolymer and mPEG-PA-PLL triblock copolymer are presented in Figure 1. ^1^H-NMR spectrum (TFA-d_6_) of mPEG-PA displayed the characteristic peaks at δ_d_ 1.51 ppm (–COCH(CH_3_)NH–), δ_a_ 3.56 ppm (–CH_2_CH_2_OCH_3_–), δ_b_ 3.89 ppm (–OCH_2_CH_2_–), and δ_c_ 4.75 ppm (–COCH(CH_3_)NH–). Then, poly(Cbz-l-Lysine) (PLL(Z)) was synthesized with an initiator, isopropylamine, by ROP of Cbz-l-Lysine-carboxyanhydride (NCA). The ^1^H-NMR spectrum (TFA-d_6_) of PLL(Z)–NH_2_ displayed characteristic peaks at δ_a_ 1.28 ppm (–CH(CH_3_)_2_NH–), δ 1.91-2.12 ppm (–NHCH_2_(CH_2_)_3_CH_2_– of the side chain), δ_b_ 4.71 ppm (–COCHNH_2_), δ_f_ 3.32 ppm (–(CH_2_)_3_CH_2_NH– of the side chain), δ_g_ 5.53 ppm (–OCH_2_C–) and δ_h_ 7.48 ppm (protons on benzyl group, Cbz). Finally, mPEG-PA copolymer and PLL(Z) was coupled by NHS–ether reaction. The mPEG-PA-PLL(Z) reacted with 33% HBr in TFA to remove Cbz groups in PLL. The ^1^H-NMR spectrum (TFA-d_6_) of the mPEG-PA-PLL triblock copolymer showed the disappearance of the characteristic peaks at δ 5.53 and 7.48 ppm (protons on Cbz) to indicate that mPEG-PA-PLL triblock copolymers were obtained. The number average molecular weight (Mn) of mPEG-PA-PLL could be calculated by integrating the proton signals of the ^1^H NMR spectra.

**Figure 2:**
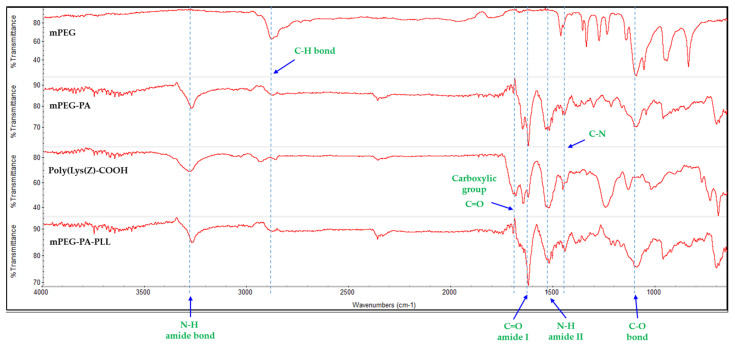
FT-IR spectrum of mPEG-NH_2_, mPEG-PA, PLL(Z)-COOH, and mPEG-PA-PLL.

The FTIR spectra of mPEG-NH_2_, mPEG-PA, PLL(Z)-COOH, and mPEG-PA-PLL are illustrated in Figure 2. The FTIR spectrum of PLL(Z)-COOH showed characteristic peaks at 1522 cm^−1^ (C=O of amide I), 1647 cm^−1^ (N-H of amide II), and 1683 cm^−1^ (C=O of carboxylic group), respectively, indicating the conjugation and modification of PLL(Z). The FTIR spectrum of mPEG-PA-PLL showed the existence of amide I at 1507 cm^−1^, amide II at 1626 cm^−1^, and a C-O bond at 1073 cm^−1^, indicating that the conjugation of mPEG-PA-PLL was successful.

In this study, two triblock copolymers with different PLL chain lengths were synthesized and are shown in Table 1. The designed number of l-Ala units in mPEG-PA diblocks copolymer was 28, but the actual number was higher than the original. In the ring-opening polymerization of NCA-l-Lysine(Z), the designed numbers of l-Lysine units in PLL(Z) polymers were 10 and 20, but the actual numbers in the received polymers were 9 and 19. Then, mPEG-PA and PLL(Z)-COOH were directly coupled by an NHS ester to synthesize polypeptide triblock copolymer (mPEG-PA-PLL(Z)). After deprotection of the Cbz groups in PLL(Z), the actual number of l-Lysine was less than the original because of acid degradation.

**Figure 3:**
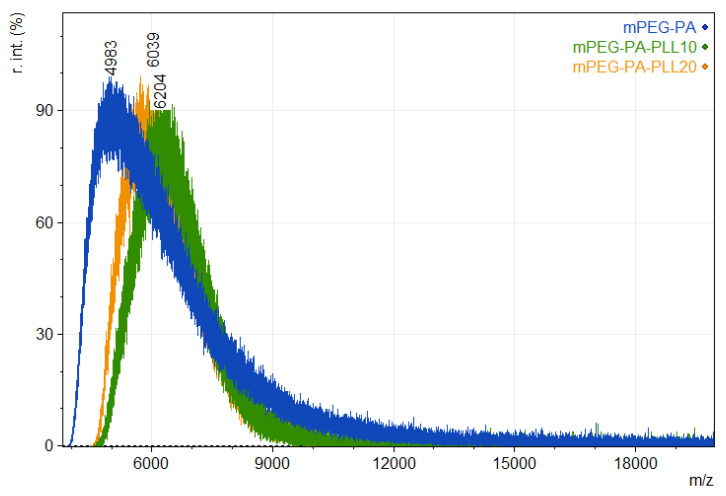
The MALDI-TOF spectra of mPEG-PA and mPEG-PA-PLL with different PLL length.

As shown in Table 1, the molecular weights of mPEG-PA-PLL measured by MALDI-TOF did not exactly match the data calculated from ^1^H-NMR. However, the MALDI-TOF spectra of mPEG-PA-PLL showed a monomodal curve, as shown in Figure 3. This result indicated mPEG-PA-PLL was a copolymer, not a blend. Due to different ionization efficiency of polymers, the molecular weight distributions measured by MALDI-TOF were affected by the selected matrix, cationization reagent, solvent, mixing ratio of cationization reagent to polymer, mixing ratio of matrix to polymer, and laser intensity [36]. Therefore, the molecular weights of the copolymers calculated from ^1^H-NMR spectra were used in this study. Overall, the above results confirmed that mPEG-PA-PLL was successfully produced.

### 2.2. Sol–Gel Phase Transition and Rheological Properties

**Figure 4:**
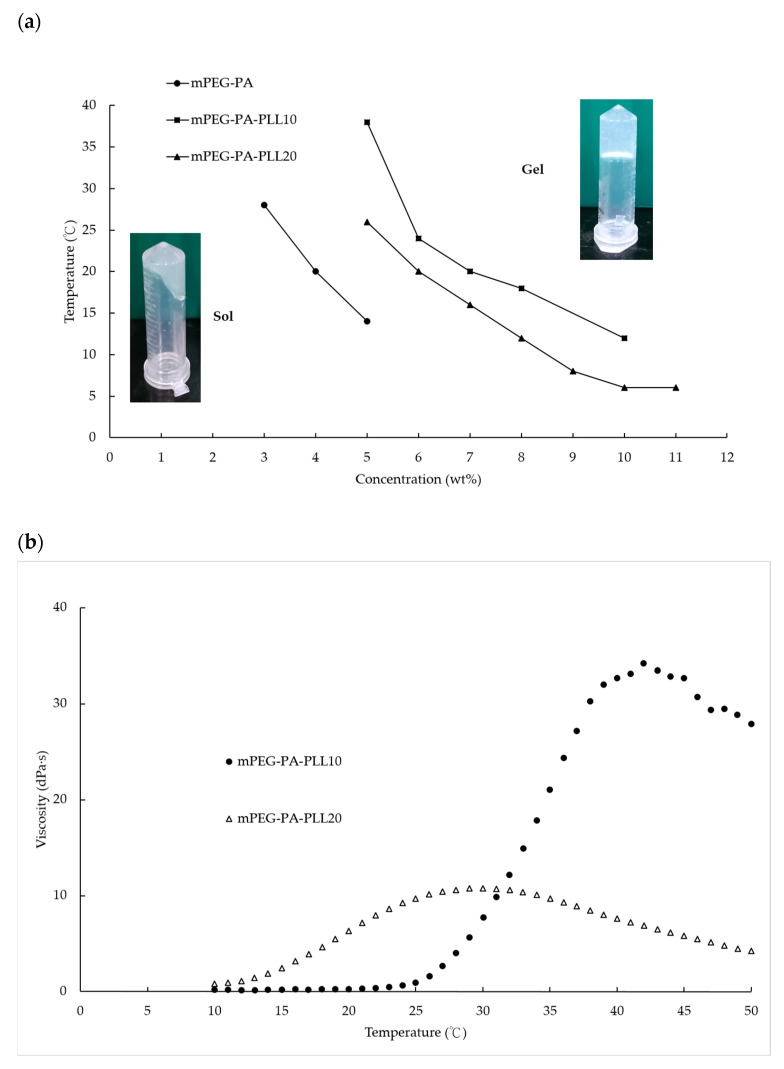
(**a**) The sol–gel transition diagram of mPEG-PA and mPEG-PA-PLL with different PLL length. (**b**) The viscosity of 5 wt% mPEG-PA-PLL with increasing temperature.

The sol–gel transition phase diagram is illustrated in Figure 4a. mPEG-PA and mPEG-PA-PLL copolymers were prepared in deionized water at the different concentrations from 3% to 11% and mixed overnight at 4 °C. The results showed a higher gelation temperature of mPEG-PA-PLL than that of mPEG-PA, due to lysine’s ionic, thus hydrophilic, nature. However, as the l-lysine repeated unit increased, the gelation temperature of mPEG-PA-PLL noticeably decreased at the same concentration because of its large molecular weight. The above result was also consistent with the rheological test, as shown in Figure 4b. The highest viscosity value of mPEG-PA-PLL_10_ and mPEG-PA-PLL_20_ was 34.2 dPa × s at 42 °C and 10.7 dPa × s at 30 °C, respectively.

### 2.3. SEM Observation

**Figure 5:**
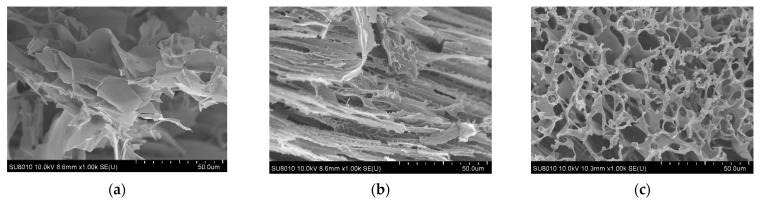
SEM images of lyophilized mPEG-PA and mPEG-PA-PLL hydrogel (5 wt%). (**a**) mPEG-PA; (**b**) mPEG-PA-PLL_10_; (**c**) mPEG-PA-PLL_20_.

The structures of mPEG-PA and PEG-PA-PLL hydrogel were observed by SEM as shown in Figure 5. As the number of l-Lysine unit increases, the microstructure of the hydrogel changed from a plate-like structure to a fibrous network. Although the internal morphology of the lyophilized hydrogel may be different with the swelling hydrogel, it is still helpful to realize the details of internal microstructure [19]. This porous structure enables a hydrogel’s ability to act as the carriers for drug delivery.

### 2.4. Biocompatibility Testing

**Figure 6:**
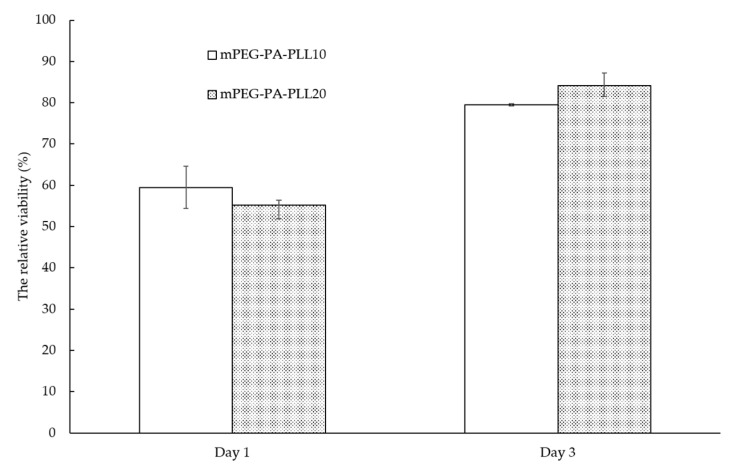
The relative viability of mPEG-PA-PLL with a different PLL length (*n* = 3). The white bar is mPEG-PA-PLL_10_ and the black dot bar is mPEG-PA-PLL_20_.

**Figure A1:**
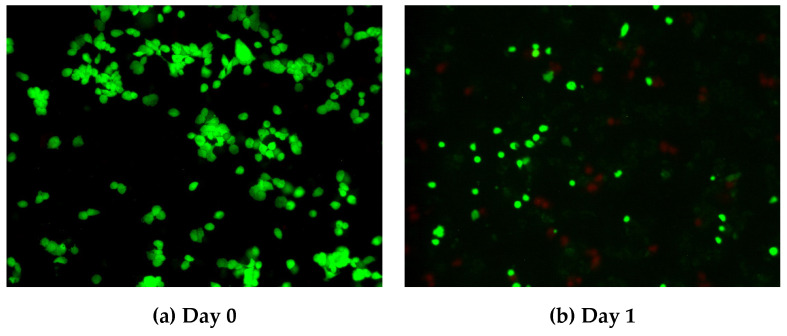
The live/dead staining images of 293T cell. The live cells stained green and dead cells were red. (**a**) control; (**b**) co-culture with mPEG-PA-PLL after Day 1.

The hydrogels must be checked not to cause any toxicity for use in biomedical applications. In the previous study [17,18], mPEG-PA had a good biocompatibility. Therefore, 293T, a human embryonic kidney cell, was used to investigate cell biocompatibility for mPEG-PA-PLL hydrogel. The cell lines were incubated in an atmosphere containing 5% CO_2_ at 37 °C for 1 day and 3 days, respectively, and fresh medium was provided on the second day. As shown in Figure 6, after 1 day of incubation, the cell viability of 293T was only approximately 59% and 55% for mPEG-PA-PLL_10_ and mPEG-PA-PLL_20_, respectively. As shown in Figure A1, the live/dead staining images also displayed the similar results. However, after 3 days of incubation, the cell viability of 293 increased to approximately 79% and 84% for mPEG-PA-PLL_10_ and mPEG-PA-PLL_20_, respectively. The past several studies showed that PLL caused the rupture of cell membranes because of its strong cation on the side chain [37,38,39]. These results also indicated that the toxicity of the mPEG-PA-PLL triblock copolymer was consistent with previous studies of PLL. However, the previous in vivo studies actually demonstrated no toxic effect of PLL in mice or rat [23,38]. After culture medium renewal on day 2, 293T cell line could grow normally and showed non-toxicity effect. In summary, mPEG-PA-PLL triblock copolymer could potentially be used in oral delivery.

### 2.5. In Vitro Biodegradability

**Figure 7:**
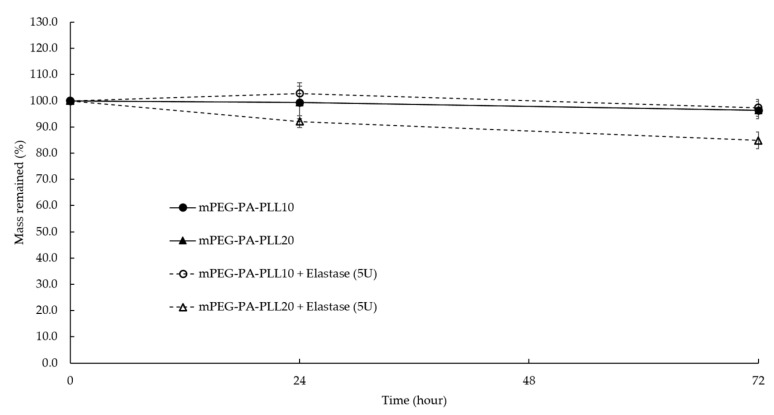
The degradation profiles of mPEG-PA-PLL with different PLL length in Tris-HCl buffer (pH 8.5) with or without 5 U/mL elastase (*n* = 4).

The in vitro degradation of the mPEG-PA-PLL hydrogel within 3 days is presented in Figure 7. The elastase in a Tris-HCl buffer with pH 8.5 (the optimum pH for elastase) was used, because elastase could catalyze the cleavage of carboxyl groups in hydrophobic amino acids, such as alanine [40]. Both hydrogels showed no clear change in Tris-HCl buffer without an enzyme over 3 days. This indicated that mPEG-PA-PLL would be stable in normal state. However, the mPEG-PA-PLL_20_ hydrogels in a Tris-HCl buffer with enzymes showed a slow degradation within 3 days. In the previous study [18], mPEG-PA hydrogel had a fast degradation in a PBS buffer with an enzyme. According to SEM imagines, the mPEG-PA-PLL hydrogel has a more complicated network structure than mPEG-PA. Elastase should not easily enter the interior of the hydrogel. Aside from this, elastase could be affected by the charge on the side chain of lysine to lose its activity. Overall, these results suggested that mPEG-PA-PLL hydrogel should exhibit a slower degradation because of the charge interaction with enzymes and the more complicated 3D microstructure.

### 2.6. Encapsulation Efficiency and In Vitro Drug-Release Studies

Calcitonin was used as a model drug for encapsulation efficiency and the release study. The encapsulation of calcitonin in a mPEG-PA-PLL hydrogel with a different chain length of PLL was released in specific time intervals with different pH values of the release medium. Generally, 0.1 N HCl and potassium phosphate-buffered solution (pH 6.8) were chosen as pharmaceutical release mediums to mimic the environment in the gastrointestinal tract. The encapsulation efficiencies (EE) and loading content of the mPEG-PA-PLL hydrogel were measured by HPLC as shown in Table 2. The results indicated that the EE of mPEG-PA-PLL hydrogels were over 96% and recommended the mPEG-PA-PLL hydrogel encapsulated calcitonin as a reservoir to apply in drug delivery.

This is the equation of the encapsulation efficiency (EE):

(1) EE=(Experimental drug loading)/(Theoretical drug loading) × 100%.

This is the equation of the loading content (LC):

(2) LC=(Experimental drug loading)/(Theoretical hydrogel loading) × 100%.

**Figure 8:**
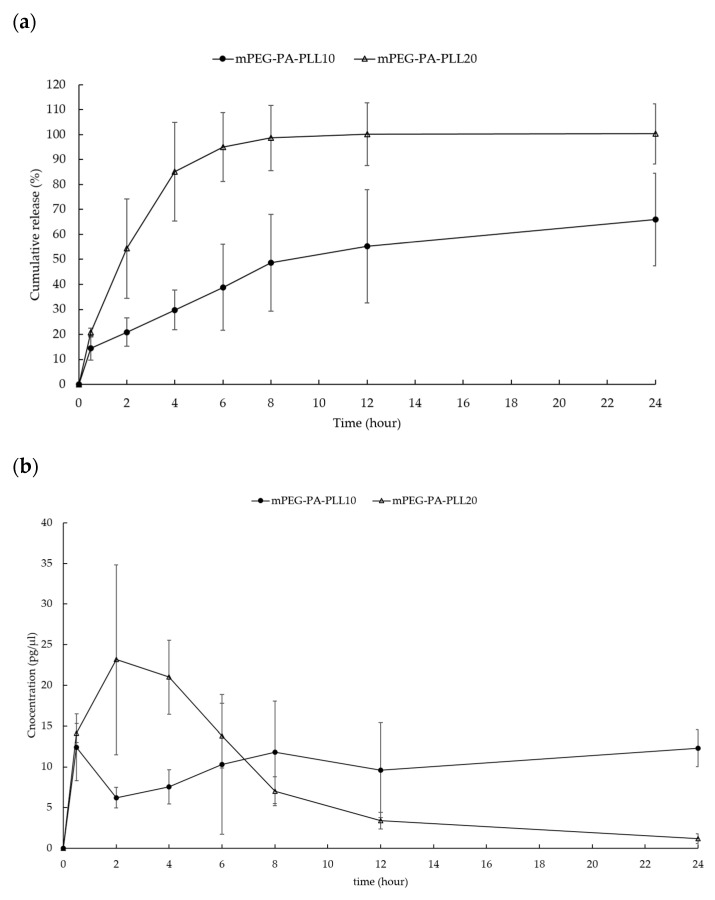
In vitro drug release profiles of mPEG-PA-PLL with different PLL length (*n* = 3). (Mean ± SD) (**a**) cumulative drug release profiles within 24 h; (**b**) drug concentrations data in the release solution taken at specific time points. The profile in (**a**) was compiled from the data points in (**b**).

In vitro drug release profiles of mPEG-PA-PLL hydrogel during 24 h are presented in Figure 8a. The drug release of mPEG-PA-PLL hydrogels had a burst effect in 0.1 N HCl solution within the first half hour and were 14.5 ± 4.8% and 20.7 ± 1.7%, respectively. However, the swelling capability of pH-sensitive hydrogels was affected by several factors such as ionic charge, the pK_a_ or pK_b_ values of ionizable groups, degree of ionization, hydrophilicity, polymer concentration, and pH of the swelling medium [15]. In the buffer solution of pH 6.8, the calcitonin contents released from the hydrogels within 24 h were 65.9 ± 18.5% and 100.3 ± 12.1%, respectively. The drug release from the hydrogel of mPEG-PA-PLL_10_ was relatively slower than mPEG-PA-PLL_20_ after 24 h; therefore, the latter shows a better bioavailability, considering the resident time of the dosage form in the GI tracts. The results suggested that this difference may be affected by a degree of ionization of the polymer and the pH of the swelling medium.

As shown in Figure 8b, mPEG-PA-PLL_10_ hydrogel continued to steadily release the drug, but the mPEG-PA-PLL_20_ release rate decreased with time. These results also showed that the drug release rate of mPEG-PA-PLL hydrogel depended on the number of l-Lysine units in PLL. There are two models to explain the release rate in a reservoir. One is a zero-order release with a constant rate of drug released. Another is a first-order release being proportional to the drug concentration [41]. Due to its constant rate, the release profile of mPEG-PA-PLL_10_ should be a zero-order release. However, a first-order release is difficult to conceptualize when using a basic theory.

The release of the drug that follows first-order kinetics can be expressed by the equation:

(3) dC/dt=−kC

where C is the concentration of drugs within hydrogels, and k is the first-order release constant expressed in units of time^−1^.

Equation (3) can be express as:

(4) $$\log C=\log C_{0}-\mathrm{kt}/2.303$$

where C_0_ is the initial concentration of the drug within hydrogels, k is the first-order rate constant, and t is the time [42,43].

**Figure A2:**
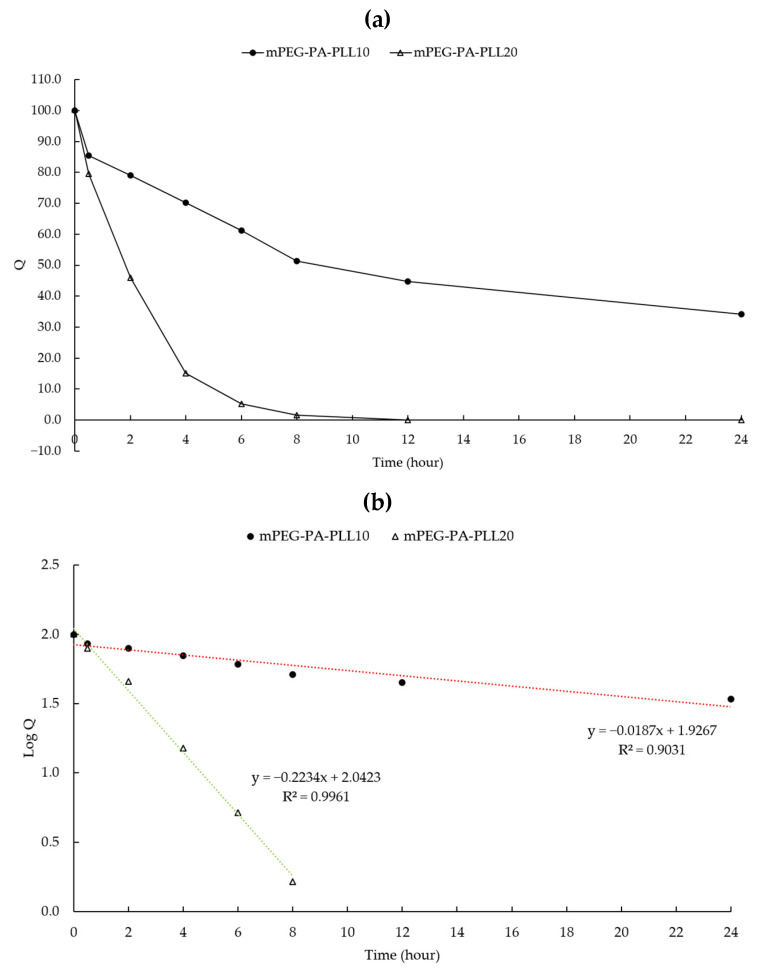
Cumulative release of calcitonin from the hydrogels. (**a**) Remaining drug percentage (Q) versus time, (**b**) Log Q versus time.

As shown in Figure A2, the experimental data obtained are plotted as a log cumulative percentage of the drug remaining versus time, which yields a straight line with a slope of −k/2.303. The coefficient of determination (R^2^) values of mPEG-PA-PLL_10_ and mPEG-PA-PLL_20_ were 0.9031 and 0.9961, respectively. Therefore, the release model of the mPEG-PA-PLL_20_ hydrogel suggested that it should be a first-order process. Its first-order rate constant is 0.514 h^−1^. The sustained release of calcitonin indicates that the mPEG-PA-PLL hydrogels have a potential for oral drug delivery, especially the mPEG-PA-PL_20_ hydrogel. Calcitonin could be fully released from the mPEG-PA-PLL_20_ hydrogel after 24 h. Therefore, this dosage form can achieve better bioavailability.

## 3. Conclusions

In this study, a newly designed triblock copolymer, mPEG-PA-PLL was successfully synthesized and applied to a hydrogel system. The characteristics of mPEG-PA-PLL triblock copolymer were confirmed by ^1^H-NMR, FTIR, and MALDI-TOF. This hydrogel system had a thermo-sensitive property with a low gelling concentration. Because of 3D microarchitecture, the hydrogel was a good reservoir for the peptide drug. In addition, the calcitonin-loaded hydrogel based on mPEG-PA-PLL also performed a pH-responsive drug release behavior. The drug release study showed that it could be suitable for oral delivery due to a sustained-release for one day. In medical use, calcitonin injection is subcutaneously or intramuscularly administered. The recommended dosage is 100 I.U. daily or 50 I.U. twice daily for 2 to 4 weeks. (100 I.U. is equivalent to approximately 15 micrograms). The oral dosage form developed here is more convenient and could improve patient compliance. The mPEG-PA-PLL hydrogel has a low gelling concentration so as to reduce polymeric material use and has a good encapsulation efficiency for various compounds, including peptides or proteins. Overall, the mPEG-PA-PLL hydrogel has the potential to be a new approach for macromolecule delivery.

## 4. Materials and Methods

### 4.1. Materials

Poly(ethylene glycol) methyl ether (mPEG, Mn = 2000), l-alanine, sodium hydroxide, and potassium phosphate, monobasic anhydrous were purchased from Sigma-Aldrich (St. Louis, MO, USA). Calcitonin salmon was obtained from ChemScene (Monmouth Junction, NJ, USA). Succinic anhydride, 4-(Dimethylamino)pyridine (DMAP), trimethylamine (TEA), isopropylamine, *N*′,*N*′-Dicyclohexycarbodiimide (DCC), *N*-Hydroxysuccinimide (NHS), trifluoroacetic acid-d (TFA-d), trifluoroacetic acid (TFA), hydrogen bromide (33% HBr/acetic acid), and tetramethylammonium hydroxide pentahydrate were purchased from Alfa Aesar (Wall Hill, MA, USA). Nepsilon-Carbobenzyloxy-l-lysine (98%) (Cbz-l-Lysine) was obtained from ACROS (Morris Plains, NJ, USA). Diethyl ether, n-hexane, ammonium hydroxide (28~30%), and ethanol (95%) were purchased from Echo Chemicals (Toufen, Miaoli, Taiwan). Dimethyl sulfoxide (DMSO), and acetonitrile (ACN) were obtained from J.T. Baker (Radnor, PA, USA). Toluene and tetrahydrofuran (THF) were obtained from TEDIA (Fairfield, OH, USA), and chloroform, dichloromethane (DCM), and *N*,*N*-dimethylformaide (DMF) were purchased from AVANTOR (Center Valley, PA, USA). Hydrochloric acid (37%) and phosphorus acid (85%) were obtained from Merck (Burlington, MA, USA). Tris(hydroxymethyl)aminomethane (TRIS) and tris(hydroxymethyl)aminomethane hydrochloride (TRIS HCl) were purchased from Apollo (Bredbury, Stockport, UK). Elastase was purchased from CHUMEIA (Hsinchu, Taiwan). All solvents were dried over CaH_2_ before used.

### 4.2. Synthesis of mPEG-PA-PLL

#### 4.2.1. Synthesis of mPEG-PA Diblock Copolymer

The synthesis of mPEG-PA was prepared as described in a previous study [18]. The mPEG-PA diblock polymer was synthesized with mPEG-NH_2_ as initiator by ring-opening polymerization (ROP) of l-alanine-*N*-carboxyanhydride (NCA-Ala).

#### 4.2.2. Synthesis of Cbz-l-lysine-*N*-carboxyanhydride (NCA-Lys(Z)) and Monocarboxylic Acid-PLL(Z)

The synthesis of NCA-Lys(Z) was similar with NCA-Ala. Cbz-l-Lysine (10 g, 35.7 mmol) and triphosgene (6.35 g, 21.4 mmol) were dissolved in anhydrous THF (150 mL) and gently stirred at 50 °C under the nitrogen flux for 3 h. Then, the mixture was condensed to a final volume of about 15 mL through rotary evaporation. Finally, an excess of n-hexane was added the mixture to obtain NCA-Lys(Z). The precipitate was collected by filtration and dried under vacuum for 2 days at room temperature.

The poly(Lys(Z)), PLL(Z) polymer was synthesized with isopropylamine as initiator by ROP of NCA-Lys(Z). NCA-(Lys(Z)) (10 or 20 mmole) was dissolved in anhydrous DMF (30 mL), and then isopropylamine (1 mmole) was added to synthesize PLL(Z) at room temperature for 72 h under the nitrogen flux. An excess of anhydrous diethyl ether was added to obtain PLL(Z). The precipitate was collected by centrifugation (3000× *g* for 5 min) and dried under vacuum for 2 days at room temperature.

Monocarboxylic acid of PLL(Z) was conducted with PLL(Z) (1 mmole), succinic anhydride (1.2 mmole), DMAP (1.2 mmole), and TEA (1.2 mmole) in DMF at room temperature for 24 h under the nitrogen flux. An excess of anhydrous diethyl ether was also added to obtain PLL(Z)-COOH. Then, the precipitate was collected by centrifugation (3000× *g* for 5 min) and dried under vacuum for 2 days at room temperature.

#### 4.2.3. Synthesis of mPEG-PA-PLL Triblock Copolymer

To synthesize mPEG-PA-PLL(Z) triblock copolymer, mPEG-PA (1 mmole), PLL(Z)-COOH (1 mmole), NHS (1.2 mmole), and DCC (1.2 mmole) were dissolved in anhydrous DMF (50 mL) at room temperature for 72 h under the nitrogen flux. An excess of anhydrous diethyl ether was added to obtain mPEG-PA-PLL(Z). Then, the precipitation was collected by centrifugation (3000× *g* for 5 min) and dried under vacuum for 2 days at room temperature.

mPEG-PA-PLL(Z) was dissolved in TFA, and 33% HBr/acetic acid was added to the deprotection of Cbz group on Lys at room temperature for 1 h. An excess of anhydrous diethyl ether was added to obtain mPEG-PA-PLL. Then, the precipitate was collected by centrifugation (3000× *g* for 5 min) and solubilized in DMSO. After dialyzing against water for 3 days (molecular weight cut-off (MWCO): 3.5 kDa), the product was collected via lyophilization and stored under vacuum.

### 4.3. Characterization of mPEG-PA-PLL Copolymers and Polypeptide Hydrogels

#### 4.3.1. Fourier Transform Infrared Spectroscopy (FT-IR)

The functional group identification of mPEG-PA-PLL co-polymer was carried out by Fourier transform infrared spectroscopy (FT-IR). Infrared spectra of co-polymers were performed using an FTIR spectrometer (NicoletTM iS50, Thermo Fisher Scientific, Waltham, MA, USA). Spectra were collected 32 times in the wavelength range from 650 to 4000 cm^−1^, and the resolution was 1 cm^−1^.

#### 4.3.2. ^1^H Nuclear Magnetic Resonance (^1^H-NMR) Spectroscopy

The ^1^H NMR spectra of the 3 wt% copolymer solution in TFA-d was obtained using a NMR spectrometer (Varian Unity INOVA 500, Palo Alto, CA, USA) at the Instrument Center of National Tsing Hua University and was used to verify the chemical structure of the copolymers.

#### 4.3.3. MALDI-TOF Mass Spectroscopy

A MALDI-TOF mass spectroscopy (Autoflex III smartbeam LRF200 CID, Bruker Daltonics, Billerica, MA, USA) from the Department of Chemistry of National Tsing Hua University was used. Copolymers were dissolved in DMF at the concentration of 2%. The α-cyano-4-hydroxycinnamic acid (HCCA) was selected as a matrix solution by mixing 1 μL sample solution on ground-steel MALDI target plate with 1 μL HCCA solution (53 mM, in ACN and water with 0.1% TFA). Once the samples were dry, the target plate was analyzed by MALDI-TOF MS.

#### 4.3.4. Sol–Gel Phase Transition and Rheological Properties

The sol–gel transition behavior of copolymers prepared at various concentrations was observed using the test tube inversion method. Samples were prepared in 2 mL Eppendorf (EP) tube at 3 to 10 wt% in deionized water and placed in a tube rotator overnight at 4 °C until the solutes were completely dissolved. The inverted Eppendorf tube was used at a temperature range of 4–50 °C, at increments of 2 °C. For temperature equilibrium, the sample was placed in dry-bath incubator for 10 min. The gelation point was designated as the temperature at which the solution stopped flowing.

The viscosity of the polymer solutions was measured at different temperatures by using rheometer (AR2000ex system, TA Instrument, New Castle, DE, USA). The sample solution was prepared at 5 wt% and allowed to fully equilibrate at 4 °C overnight. Approximately 500 µL of sample solution was loaded on a prechilled test plate at 10 °C on a system with a 25 mm parallel plate geometry and gap of 1 mm. The rheometer was conducted using a dynamic temperature from 10 to 50 °C in 15 min. A shear rate of 10% and an angular frequency of 1 rad/s were set up.

#### 4.3.5. Scanning Electron Microscopy (SEM)

Next, 5 wt% of the copolymer solution was prepared at 4 °C overnight before use. The hydrogels were frozen in liquid nitrogen and lyophilized for 72 h. Then, the hydrogels were sputtered with gold before observation. The morphology of hydrogels was examined using an SEM system (JEOL JSM-7001F, Peabody, MA, USA). Briefly, hydrogels were gelled at 37 °C for 30 min and then frozen in liquid nitrogen prior to lyophilization. To investigate the cross section of hydrogels, lyophilized samples were carefully cut by using a scalpel blade. Then, the hydrogels were sputtered with gold before observation.

### 4.4. In Vitro Degradation Testing of mPEG-PA-PLL

In vitro degradation of hydrogels (100 μL) in Tris-HCl buffer (pH 8.5) and Tris-HCl buffer (pH 8.5) containing elastase (5 U/mL) was conducted. Briefly, copolymer solutions (5 wt%) were gelled in 1.5 mL Eppendorf tube at 37 °C for 30 min prior to adding 1 mL of Tris-HCl buffer or Tris-HCl buffer with elastase. The Eppendorf tube was removed from incubator at each time point and at 37 °C; the residual was weighted after washing twice with the de-ionized water and lyophilizing for 2 days.

### 4.5. Biocompatibility

Human embryonic kidney cells (293T) were used for in vitro cytotoxicity test. The cytotoxicity test of the hydrogels was carried out by MTT assays. The cytotoxicity test was similar to the previous study [11,13]. The 293T cell line was loaded onto a 24-well transwell plate at concentration of 8 × 10^3^ cells per well with DMEM media supplemented with 10% fetal bovine serum (FBS) and 1% antibiotic-antimycotic. The copolymer solutions were prepared in DMEM media at the concentration of 5 wt%. Then, 100 μL of the copolymer solutions were loaded onto the transwell insert to gel at 37 °C and overlaid with 1 mL of DMEM media. Subsequently, every well was treated with 50 µL of MTT agent (5 mg/mL in PBS buffer) and incubated for 4 h, before 200 μL of DMSO was added to stop the reaction. The solution was then collected and analyzed. The absorbance was measured by using an ELISA Reader (Bio-Tek Synergy HT, Winooski, VT, USA) at 570 nm. All assays were repeated in triplicate for each sample.

This is cell viability of an equation:

(5) $$\mathrm{Cell}\;\mathrm{viability}=\frac{\mathrm{Absorbance}\;\mathrm{of}\;\mathrm{cells}\;\mathrm{with}\;\mathrm{hydrogel}}{\mathrm{Absorbance}\;\mathrm{of}\;\mathrm{negative}\;\mathrm{control}\;\mathrm{cells}\;}\;\times \;100\%.$$

### 4.6. Methodological Study of HPLC

HPLC (Chromaster, Hitachi, Tokyo, Japan) with a column (COSMOSIL Packed Column, 5C_18_-AR-II, 4.6 mm × 250 mm) was conducted to quantify the concentration of calcitonin. Solution A: dissolve 3.26 g of tetramethylammonium pentahydrate in 900 mL of water, add 100 mL of acetonitrile, and mix; adjust with phosphoric acid to a pH of 2.5, pass through a filter of 0.22 mm, and degas. Solution B: dissolve 1.45 g of tetramethylammonium hydroxide pentahydrate in 400 mL of water, add 600 mL of acetonitrile, and mix; adjust with phosphoric acid to a pH of 2.5, pass through a filter of 0.22-mm, and degas. The flow rate was 1.0 mL/min with a detector set at 220 nm. Injection volume of the sample was 20 μL.

### 4.7. In Vitro Drug-Release Studies

Initially, 100 μg of calcitonin-loaded mPEG-PA-PLL hydrogels (10 wt%) were immersed in 1.5 mL of 0.1 N HCl solution and added to 2 mL Eppendorf (EP) tubes under the condition of 37 ± 0.2 °C and 50 rpm for 2 h. At specific time intervals (0.5 and 2 h), 1 mL of the release media was withdrawn, and an equal volume of medium was added to maintain the volume constant. Finally, the drug-loaded hydrogels were transferred into 1.5 mL of potassium phosphate-buffered solution (pH 6.8), and 1 mL of release media was collected again at specific times (4, 6, 8, 12, and 24 h) until 24 h. HPLC was determined to ascertain the calcitonin concentration. A linear calibration curve over the concentration range of 0.00391 mg/mL to 2.0 mg/mL was constructed, and the calcitonin concentration was accordingly interpolated.

## Figures and Tables

**Table 1:** Characterization of the mPEG-PA, PLL(Z), and mPEG-PA-PLL triblock copolymers.

Block Copolymer	Designed Ratio ^a^	Actual Ratio ^b^	Mn ^c^	Mz ^d^	PDI ^e^
mPEG_45_-PA_28_	1:28	1:31	4315	4983	1.060
PLL_10_(Z)	1:10	1:9	2496	-	-
PLL_20_(Z)	1:20	1:19	5435	-	-
mPEG_45_-PA_28_-PLL_10_	1:28:10	1:34:4	5343	6204	1.024
mPEG_45_-PA_28_-PLL_20_	1:28:20	1:30:14	6741	6039	1.027

^a^ The molar ratio of monomer NCA–amino acid to initiator in feeding. ^b^ The molar ratio of monomer NCA–amino acid to initiator determined by ^1^H-NMR spectra. ^c^ Determined by ^1^H-NMR spectra. ^d^ Determined by MALDI-TOF spectra. ^e^ Determined by MALDI-TOF spectra.

**Table 2:** The encapsulation efficiency and loading content of mPEG-PA-PLL (*n* = 3).

	mPEG-PA-PLL_10_	mPEG-PA-PLL_20_
Encapsulation Efficiency (EE) (%)	99.37 ± 0.03	96.80 ± 1.76
Loading content (LC) (%)	1.25 ± 0.04	1.00 ± 0.03

## Data Availability

The data presented in this study are available on request from the corresponding author. The data are not publicly available due to privacy.

## References

[1] Mitragotri S., Burke P.A., Langer R. (2014). Overcoming the Challenges in Administering Biopharmaceuticals: Formulation and Delivery Strategies. Nat. Rev. Drug Discov..

[2] Anselmo A.C., Gokarn Y., Mitragotri S. (2018). Non-Invasive Delivery Strategies for Biologics. Nat. Rev. Drug Discov..

[3] Brown T.D., Whitehead K.A., Mitragotri S. (2020). Materials for Oral Delivery of Proteins and Peptides. Nat. Rev. Mater..

[4] Zhu Q., Chen Z., Paul P.K., Lu Y., Wu W., Qi J. (2021). Oral Delivery of Proteins and Peptides: Challenges, Status Quo and Future Perspectives. Acta Pharm. Sin. B.

[5] Dubey S.K., Parab S., Dabholkar N., Agrawal M., Singhvi G., Alexander A., Bapat R.A., Kesharwani P. (2021). Oral Peptide Delivery: Challenges and the Way Ahead. Drug Discov. Today.

[6] Jash A., Ubeyitogullari A., Rizvi S.S.H. (2021). Liposomes for Oral Delivery of Protein and Peptide-Based Therapeutics: Challenges, Formulation Strategies, and Advances. J. Mater. Chem. B.

[7] Mansoor S., Kondiah P.P.D., Choonara Y.E. (2021). Advanced Hydrogels for the Controlled Delivery of Insulin. Pharmaceutics.

[8] Xu Y., Michalowski C.B., Beloqui A. (2021). Advances in Lipid Carriers for Drug Delivery to the Gastrointestinal Tract. Curr. Opin. Colloid Interface Sci..

[9] Paroha S., Chandel A.K.S., Dubey R.D. (2018). Nanosystems for Drug Delivery of Coenzyme Q10. Environ. Chem. Lett..

[10] Bera A., Singh Chandel A.K., Uday Kumar C., Jewrajka S.K. (2015). Degradable/Cytocompatible and PH Responsive Amphiphilic Conetwork Gels Based on Agarose-Graft Copolymers and Polycaprolactone. J. Mater. Chem. B.

[11] Chandel A.K.S., Kumar C.U., Jewrajka S.K. (2016). Effect of Polyethylene Glycol on Properties and Drug Encapsulation-Release Performance of Biodegradable/Cytocompatible Agarose-Polyethylene Glycol-Polycaprolactone Amphiphilic Co-Network Gels. ACS Appl. Mater. Interfaces.

[12] Sharpe L.A., Daily A.M., Horava S.D., Peppas N.A. (2014). Therapeutic Applications of Hydrogels in Oral Drug Delivery. Expert Opin. Drug Deliv..

[13] Qiu Y., Park K. (2001). Environment-Sensitive Hydrogels for Drug Delivery. Adv. Drug Deliv. Rev..

[14] Schmaljohann D. (2006). Thermo- and PH-Responsive Polymers in Drug Delivery. Adv. Drug Deliv. Rev..

[15] Rizwan M., Yahya R., Hassan A., Yar M., Azzahari A.D., Selvanathan V., Sonsudin F., Abouloula C.N. (2017). PH Sensitive Hydrogels in Drug Delivery: Brief History, Properties, Swelling, and Release Mechanism, Material Selection and Applications. Polymers.

[16] Lin S.J., Chan Y.C., Su Z.C., Yeh W.L., Lai P.L., Chu I.M. (2021). Growth Factor-Loaded Microspheres in MPEG-Polypeptide Hydrogel System for Articular Cartilage Repair. J. Biomed. Mater. Res.-Part A.

[17] Anggelia M.R., Cheng H.Y., Wen C.J., Wang A.Y.L., Lin C.H. (2019). A Mixed Thermosensitive Hydrogel System for Sustained Delivery of Tacrolimus for Immunosuppressive Therapy. Pharmaceutics.

[18] Lin H.C., Chen C.Y., Kao C.W., Wu S.T., Chen C.L., Shen C.R., Juang J.H., Chu I.M. (2020). In Situ Gelling-Polypeptide Hydrogel Systems for the Subcutaneous Transplantation of MIN6 Cells. J. Polym. Res..

[19] Sim J., Lee H.J., Jeong B., Park M.H. (2021). Poly(Ethylene Glycol)-Poly(l-Alanine)/Hyaluronic Acid Complex as a 3d Platform for Understanding Cancer Cell Migration in the Tumor Microenvironment. Polymers.

[20] Wu I.E., Anggelia M.R., Lin S.Y., Chen C.Y., Chu I.M., Lin C.H. (2021). Thermosensitive Polyester Hydrogel for Application of Immunosuppressive Drug Delivery System in Skin Allograft. Gels.

[21] Chandel A.K.S., Shimizu A., Hasegawa K., Ito T. (2021). Advancement of Biomaterial-Based Postoperative Adhesion Barriers. Macromol. Biosci..

[22] Choi Y.Y., Jang J.H., Park M.H., Choi B.G., Chi B., Jeong B. (2010). Block Length Affects Secondary Structure, Nanoassembly and Thermosensitivity of Poly(Ethylene Glycol)-Poly(l-Alanine) Block Copolymers. J. Mater. Chem..

[23] Ayyappan J.P., Sami H., Rajalekshmi D.C., Sivakumar S., Abraham A. (2014). Immunocompatibility and Toxicity Studies of Poly-L-Lysine Nanocapsules in Sprague–Dawley Rats for Drug-Delivery Applications. Chem. Biol. Drug Des..

[24] Al-Jamal K.T., Al-Jamal W.T., Wang J.T.W., Rubio N., Buddle J., Gathercole D., Zloh M., Kostarelos K. (2013). Cationic Poly-L-Lysine Dendrimer Complexes Doxorubicin and Delays Tumor Growth in Vitro and in Vivo. ACS Nano.

[25] Chandel A.K.S., Bera A., Nutan B., Jewrajka S.K. (2016). Reactive Compatibilizer Mediated Precise Synthesis and Application of Stimuli Responsive Polysaccharides-Polycaprolactone Amphiphilic Co-Network Gels. Polymer (Guildf).

[26] Chandel A.K.S., Nutan B., Raval I.H., Jewrajka S.K. (2018). Self-Assembly of Partially Alkylated Dextran-Graft-Poly[(2-Dimethylamino)Ethyl Methacrylate] Copolymer Facilitating Hydrophobic/Hydrophilic Drug Delivery and Improving Conetwork Hydrogel Properties. Biomacromolecules.

[27] Singh Chandel A.K., Kannan D., Nutan B., Singh S., Jewrajka S.K. (2017). Dually Crosslinked Injectable Hydrogels of Poly(Ethylene Glycol) and Poly[(2-Dimethylamino)Ethyl Methacrylate]-: B-Poly(N-Isopropyl Acrylamide) as a Wound Healing Promoter. J. Mater. Chem. B.

[28] Peppas N.A., Khare A.R. (1993). Preparation, Structure and Diffusional Behavior of Hydrogels in Controlled Release. Adv. Drug Deliv. Rev..

[29] Massa S., Fouad A., Ebrahimi M., Maria P.L.S., ElAssal R., Gaudilliere D., Connelly S.T. (2021). Emerging Trends in Oral Mucoadhesive Drug Delivery for Head and Neck Cancer. Early Detection and Treatment of Head & Neck Cancers.

[30] Witten J., Samad T., Ribbeck K. (2018). Selective Permeability of Mucus Barriers. Curr. Opin. Biotechnol..

[31] Brizzolara D., Cantow H.J., Diederichs K., Keller E., Domb A.J. (1996). Mechanism of the Stereocomplex Formation between Enantiomeric Poly(Lactide)S. Macromolecules.

[32] Copp D.H., Cheney B. (1962). Calcitonin—a Hormone from the Parathyroid Which Lowers the Calcium-Level of the Blood. Nature.

[33] Foster G.V., Baghdiantz A., Kumar M.A., Slack E., Soliman H.A., MacIntyre I. (1964). Thyroid Origin of Calcitonin. Nature.

[34] Chesnut C.H., Azria M., Silverman S., Engelhardt M., Olson M., Mindeholm L. (2008). Salmon Calcitonin: A Review of Current and Future Therapeutic Indications. Osteoporos. Int..

[35] Victor S.P., Paul W., Sharma C.P. (2014). Eligen® Technology for Oral Delivery of Proteins and Peptides. Mucosal Delivery of Biopharmaceuticals.

[36] Wang Z., Zhang Q., Shen H., Yang P., Zhou X. (2021). Optimized MALDI-TOF MS Strategy for Characterizing Polymers. Front. Chem..

[37] Karabasz A., Szczepanowicz K., Cierniak A., Bereta J., Bzowska M. (2018). In Vitro Toxicity Studies of Biodegradable, Polyelectrolyte Nanocapsules. Int. J. Nanomed..

[38] Isaksson K., Åkerberg D., Posaric-Bauden M., Andersson R., Tingstedt B. (2014). In Vivo Toxicity and Biodistribution of Intraperitoneal and Intravenous Poly-l-Lysine and Poly-l-Lysine/Poly-l-Glutamate in Rats. J. Mater. Sci. Mater. Med..

[39] Isaksson K., Åkerberg D., Andersson R., Tingstedt B. (2010). Toxicity and Dose Response of Intra-Abdominally Administered Poly-l-α-Lysine and Poly-L-Glutamate for Postoperative Adhesion Protection. Eur. Surg. Res..

[40] Sadeghi-kaji S., Shareghi B., Saboury A.A., Farhadian S. (2019). Spectroscopic and Molecular Docking Studies on the Interaction between Spermidine and Pancreatic Elastase. Int. J. Biol. Macromol..

[41] Patel P., Mandal A., Gote V., Pal D., Mitra A.K. (2019). Thermosensitive Hydrogel-Based Drug Delivery System for Sustained Drug Release. J. Polym. Res..

[42] Hamman J.H., Enslin G.M., Kotzé A.F. (2005). Oral Delivery of Peptide Drugs: Barriers and Developments. BioDrugs.

[43] Jeong B., Kim S.W., Bae Y.H. (2012). Thermosensitive Sol-Gel Reversible Hydrogels. Adv. Drug Deliv. Rev..

